# Case report: A case study of neoadjuvant immunochemotherapy for locally advanced esophageal squamous carcinoma

**DOI:** 10.3389/fonc.2024.1332314

**Published:** 2024-07-04

**Authors:** Xiong Liu, Maoqi Wang, Deyuan Meng, Yuntao Tang, Qingtong Shi

**Affiliations:** ^1^ Department of Thoracic Surgery, Affiliated Hospital of Yangzhou University, Yangzhou, Jiangsu, China; ^2^ Graduate School Of Dalian Medical University, Dalian Medical University, Dalian, Liaoning, China; ^3^ The 3rd Affiliated Hospital of Chengdu Medical College, Pidu District People’s Hospital, Chengdu, Sichuan, China; ^4^ Cardiac Surgery, Affiliated Hospital of Yangzhou University, Yangzhou, Jiangsu, China

**Keywords:** esophageal cancer, neoadjuvant therapy, immunotherapy, chemotherapy, surgery

## Abstract

In locally advanced esophageal cancer, the controversy over the two traditional treatment modalities, neoadjuvant radiotherapy and neoadjuvant chemotherapy, has been unending and also challenged by the addition of neoadjuvant immunotherapy. Neoadjuvant immunotherapy has led to an increasing diversity of neoadjuvant combination treatment modalities, among which neoadjuvant immunochemotherapy has emerged, with current clinical studies initially demonstrating its efficacy and safety. We report a case of a patient with locally advanced esophageal cancer who underwent two cycles of neoadjuvant immunochemotherapy and successful surgery and achieved a pathological complete response (pCR). A 73-year-old elderly female patient presented with progressive dysphagia for more than 1 month with an Eastern Cooperative Oncology Group (ECOG) score of 1. After completing gastroscopy + pathological biopsy, chest enhanced CT, barium esophageal meal, PET-CT, and other related examinations, the clinical diagnosis was thoracic segmental esophageal poorly differentiated squamous carcinoma cT2N2M0 stage III. After a multidisciplinary discussion of the comprehensive treatment plan, two cycles of neoadjuvant therapy were given on February 16, 2023, and March 9, 2023, and the treatment plan was as follows: cisplatin 30 mg d1–3 + albumin paclitaxel 200 mg d1 and 100 mg d8 + sintilimab 200 mg d4, q3w. After the neoadjuvant therapy, the patient underwent an imaging examination. The chest enhanced CT suggested that the lesion range was significantly reduced compared with the previous scan, and mediastinal lymph nodes were partially reduced. Then, thoracoscopic radical esophageal cancer surgery was performed on April 23, 2023. pCR was achieved by pathological evaluation, and the postoperative diagnosis was thoracic segmental esophageal hypofractionated squamous carcinoma ypT0N0M0. Two cycles of adjuvant immunochemotherapy were performed after surgery on May 30, 2023, and June 21, 2023, and the regimen was as follows: cisplatin 30 mg d1–3 + albumin paclitaxel 200 mg d1 and 100 mg d8 + sindilizumab 200 mg d4, q3w. As of the latest review on March 20, 2024, the patient was not seen to have any significant postoperative complications and remains in a state of complete response (CR). Neoadjuvant immunochemotherapy has positive significance for the treatment of patients with locally advanced esophageal cancer. Whether neoadjuvant immunochemotherapy can replace neoadjuvant synchronous radiotherapy is a future direction of research, which needs to be further verified by more reliable clinical trials.

## Introduction

Esophageal cancer (EC) is a malignant tumor that poses a serious threat to human health, and its incidence has been on the rise year by year in recent years. According to 2020 GLOBOCAN Global Cancer Statistics, worldwide, the incidence rate of esophageal cancer is the seventh highest, and its mortality rate is the sixth highest, with more than 500,000 cancer-related deaths every year (1). The histologic typing of esophageal cancer varies in different regions, with esophageal adenocarcinoma predominating in Europe and the United States, and esophageal squamous cell carcinoma (ESCC) predominating in China, which is a country with a high incidence of esophageal cancer, accounting for more than 95% of the cases (2). Early esophageal cancer symptoms are not obvious, and most patients are already in the middle or late stage at the time of diagnosis. Surgery is still the main treatment for patients with locally advanced esophageal cancer, but the effect of surgery alone is poor, and the 5-year survival rate is less than 30% (3, 4). National Comprehensive Cancer Network (NCCN) and Chinese Society of Clinical Oncology (CSCO) guidelines currently recommend neoadjuvant chemotherapy (nCT) and neoadjuvant radiotherapy (nRT). nCT and neoadjuvant chemoradiotherapy (nCRT) are currently recommended by both NCCN and CSCO guidelines as the standard treatment modalities for locally advanced esophageal cancer (5, 6). However, the existing neoadjuvant (radiotherapy) chemotherapy regimens have difficulty meeting the needs of treatment for patients with locally advanced esophageal cancer, and preoperative neoadjuvant chemotherapy has a low pathological complete response (pCR) rate of no more than 10%. Neoadjuvant chemoradiotherapy improves the pCR rate compared with that of neoadjuvant chemotherapy, but it may increase the postoperative complications such as anastomotic fistula (7–9), and the 5-year recurrence rate is still as high as 40%–50% (10).

Several clinical trials have been initiated for immunotherapy of esophageal cancer (11), and immune checkpoint inhibitors (ICIs) have been approved for first-line, second-line, and postoperative adjuvant treatments of esophageal cancer (12–15); however, there are no reliable clinical studies demonstrating the benefits of neoadjuvant immunotherapy for patients with esophageal cancer. Most of the studies of neoadjuvant chemotherapy combined with immunotherapy have been phase II clinical studies, and the results have now shown an increased pCR rate compared with chemotherapy alone without a significant increase in postoperative complications (16), which provides a new idea for the treatment of patients with locally advanced esophageal cancer. In this study, we report a patient with locally advanced squamous esophageal cancer who received two cycles of neoadjuvant immunotherapy in combination with chemotherapy (later referred to as neoadjuvant immunochemotherapy) prior to surgery and then achieved a pCR on pathological evaluation after surgery.

## Case presentation

**Figure 1:**
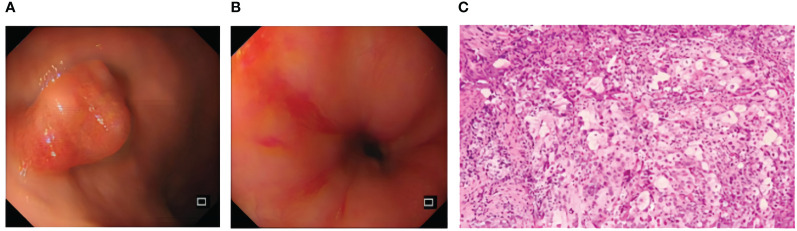
Pre-treatment examination: gastroscopy **(A, B)** and histopathological manifestation after H&E staining **(C)**.

A 73-year-old elderly female patient was previously fit with a history of surgical removal of an ovarian cyst. The patient denied a history of smoking and alcohol consumption. The patient presented to the hospital with progressive dysphagia for more than 1 month with acid reflux heartburn, retrosternal chest tightness, and discomfort with an Eastern Cooperative Oncology Group (ECOG) score of 1. Gastroscopy was performed (February 6, 2023): neoplastic growth and luminal narrowing were seen in the esophagus 23–30 cm from the incisors (**Figures 1A, B**). Gastroscopy was barely passed with poor sampling elasticity; the endoscopic diagnosis was esophageal cancer; pathological biopsy results indicated (esophageal) poorly differentiated carcinoma with a tendency to poorly differentiated squamous cell carcinoma (**Figure 1C**).

**Figure 2:**
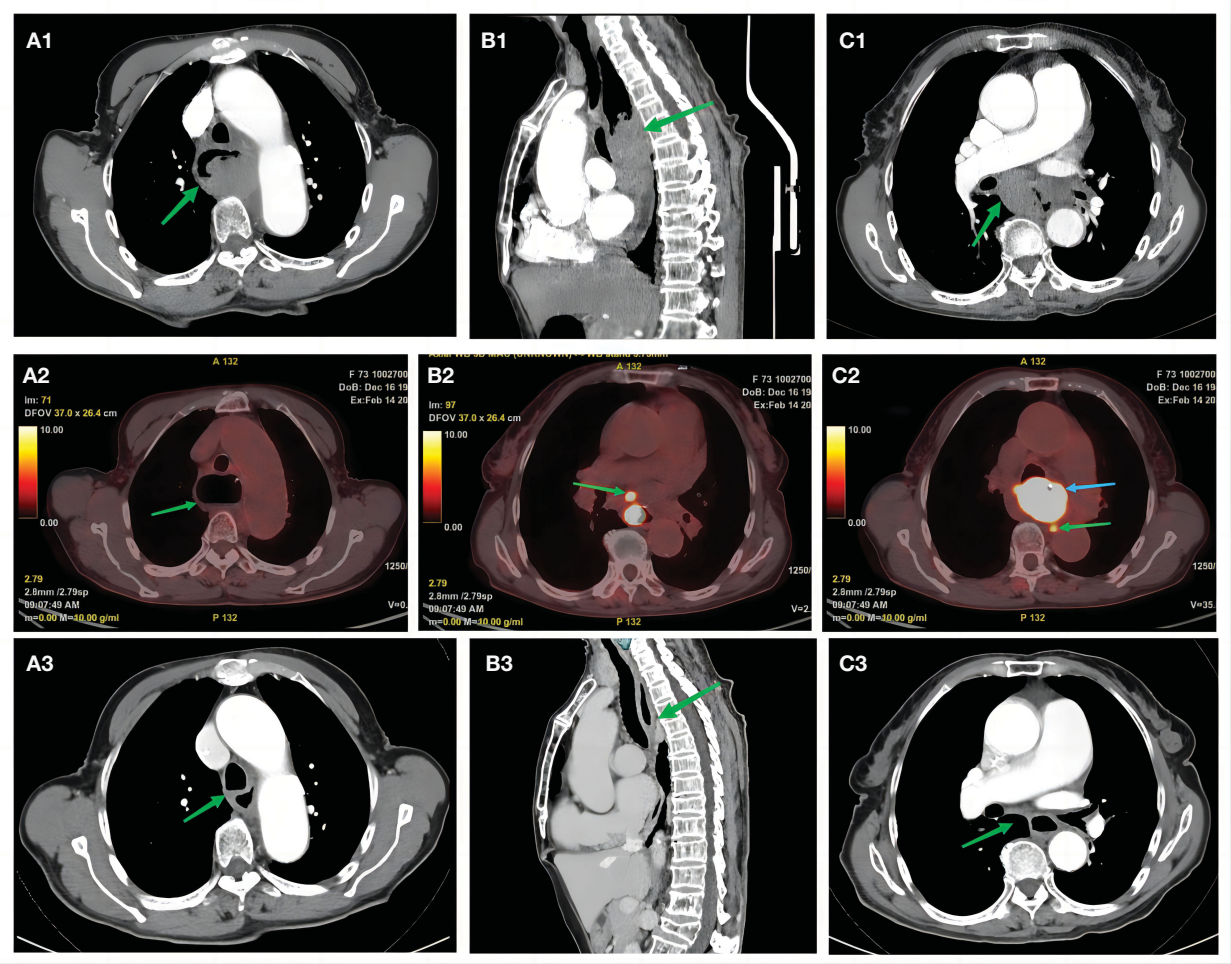
Pre-treatment examination: high-resolution computed tomography. **(A1, B1)** Green arrows indicate mid-esophageal occupations. **(C1)** Green arrow indicates an enlarged mediastinal lymph node (approximately 1.8 cm in diameter). Positron emission tomography/computed tomography examination. **(A2)** Green arrow shows dilated esophagus. **(B2)** Green arrow indicates mediastinal multiple lymph nodes with increased FDG metabolism. **(C2)** Green arrow indicates right paraesophageal hiatus lymph node with increased FDG metabolism, considered metastatic. **(C2)** Blue arrow indicates middle esophageal occupancy with increased FDG metabolism. Post-treatment examination: high-resolution computed tomography scan. **(A3, B3)** Green arrows indicate significantly shrunken esophageal cancer lesions compared with the previous scan (February 6, 2023). **(C3)** Green arrows indicate shrunken lymph nodes. FDG, fluorodeoxyglucose.

With further refinement of related examinations, chest enhanced CT revealed the following: 1) middle esophageal occupancy (thickening of the wall in the middle esophagus, nodular soft tissue mass shadow in the lumen, approximately 4.7 * 3.2 cm, moderate enhancement, and esophageal dilatation above it) (**Figures 2A1–B1**) and 2) enlarged mediastinal lymph nodes (diameter of the enlarged lymph nodes was approximately 1.8 cm) (**Figure 2C1**). PET-CT revealed the following: 1) middle esophageal occupancy with increased fluorodeoxyglucose (FDG) metabolism, considered esophageal malignant tumor [widening of the wall of the middle esophagus, narrowing of the lumen, nodular soft tissue mass shadow seen in the lumen, larger cross-section of approximately 5.0 * 3.2 cm, increased radiolucency, maximum standardized uptake value (SUV) of 43.58, and esophageal dilatation above it] (**Figure 2A**), and 2) multiple lymph nodes of the mediastinum and lymph nodes of the gastric right parietal hiatus with increased FDG metabolism, considered metastasis (mediastinum). Several lymphoid shadows with increased radioactivity uptake were seen in the mediastinum, with a maximum SUV of 10.88, located on the right side of the tracheal crest, with a long diameter of approximately 1.1 cm; a lymphoid shadow with a long diameter of approximately 0.9 cm, with increased radioactivity uptake and a maximum SUV of 21.44, was seen in the right parietal hiatus of the stomach) (**Figures 2B2, C2**). Combined with the above examinations, the patient was diagnosed with thoracic segmental esophageal poorly differentiated squamous carcinoma cT2N2M0 stage III [according to the American Joint Committee on Cancer (AJCC) 8th edition TNM staging system]. The patient’s feeding obstruction was obvious, and a nasogastric nutritional tube was placed after admission. After the thoracic surgery department evaluated the difficulties of surgery, the multidisciplinary consultation decided to carry out pre-surgical neoadjuvant treatment, and the first and second cycles of neoadjuvant immunochemotherapy were carried out on February 16, 2023, and March 9, 2023, with the following regimen: cisplatin 30 mg d1–3 + albumin paclitaxel 200 mg d1 and 100 mg d8 + sintilimab 200 mg d4, q3w. After two cycles of neoadjuvant immunotherapy, the patient underwent imaging examinations; enhanced CT of the chest suggested that after treatment of middle esophageal cancer, the extent of the lesion was significantly reduced compared with that of the previous scan (February 6, 2023) (the wall of the middle esophagus was slightly thickened with mild enhancement, and the lumen of the tube did not show obvious narrowing); the mediastinal lymph nodes were partially reduced (slightly larger lymph node shadows, multiple flattened and long, were seen in the mediastinum) (**Figures 2A3, B3, C3**).

**Figure 3:**
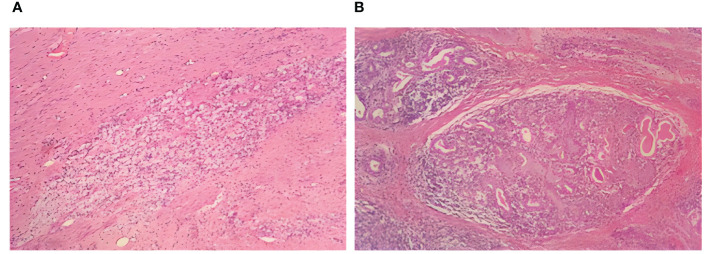
Postoperative examination: postoperative histopathological manifestations of H&E staining. **(A)** Foam-like histiocytes. **(B)** Multinucleated giant cells.

Partial relief (PR) was assessed to be achieved according to the solid tumor efficacy evaluation criteria Response Evaluation Criteria in Solid Tumors (RECIST) 1.1. Contraindications to surgery were excluded, and radical thoracoscopic esophageal cancer surgery was performed on April 23, 2023, which progressed smoothly. Postoperative pathological findings (**Figures 3A, B**) were as follows: 1) (thoracic esophagus) microscopic examination showed a large number of foam-like histiocyte hyperplasia, multinucleated giant cell reaction, epithelioid granuloma formation, lymphocytic infiltration with lymphoid hyperplasia, interstitial fibroblastic tissue hyperplasia, and localized calcium salt deposition; 2) no cancerous cells were found in the upper margins of the esophagus sample sent for examination, and the upper and lower margins of the esophagus were removed from the tissue by self-sampling; 3) two peri-esophageal lymph nodes, one lymph node of the 15th group, one lymph node of the left laryngeal reentry, and the right laryngeal reentry tissue were not found to have residual cancer cells. After pathological evaluation, the patient’s surgical specimen and sampled lymph nodes were negative, she achieved pCR and R0 resection, and she was diagnosed with thoracic segmental esophageal hypofractionated squamous carcinoma ypT0N0M0 postoperatively.

The patient’s postoperative esophageal recovery was possible, her general condition was good, and no obvious postoperative complications were seen. For comprehensive assessment of the condition, two cycles of postoperative adjuvant immunochemotherapy were performed on May 30, 2023, and June 21, 2023, with the following regimen: cisplatin 30 mg d1–3 + albumin paclitaxel 200 mg d1 and 100 mg d8 + sintilimab 200 mg d4, q3w. As of the latest review on March 20, 2024, the patient was still in a state of complete response (CR), with no tumor recurrence or metastasis.

## Discussion

With the advent of the era of tumor immunotherapy, the addition of immune checkpoint inhibitors to neoadjuvant chemotherapy has become a proven therapeutic strategy. The combination of chemotherapy and immunotherapy has certain synergistic effects: on the one hand, chemotherapy induces immunogenic death of tumor cells, releases tumor antigens, and eliminates the suppression of the immune system by cancer cells; on the other hand, effector T cells in the tumor microenvironment can enhance the efficacy of chemotherapeutic drugs by weakening the chemotherapeutic resistance mediated by the basal cells so that the combination of the two can produce the effect of “1 + 1 > 2” (17). At present, neoadjuvant immunotherapy combined with chemotherapy or radiotherapy is still in the exploratory stage, and the patient with esophageal cancer in this study underwent neoadjuvant immunochemotherapy and obtained good therapeutic effects, with no recurrence so far. The specific treatment modality of neoadjuvant immunochemotherapy for esophageal cancer is still inconclusive, so we summarized several important clinical trials of neoadjuvant immunochemotherapy, including three preliminary studies (18–20) and nine phase II clinical studies (21–30) (for details, see **Table 1**), which confirmed the efficacy and safety of the neoadjuvant immunotherapy in patients with locally advanced esophageal cancer, shrinking the tumor, lowering the stage, and effectively reducing the degree of metastasis of subclinical lesions, as well as increasing the rate of surgical resection without a significant increase in postoperative complications. Neoadjuvant immunochemotherapy has positive significance in the treatment of patients with locally advanced esophageal cancer, and whether it is expected to improve the survival of patients with locally advanced esophageal cancer needs to be further verified in a large-sample phase III clinical study.

**Table 1:** Summary of important clinical trials of neoadjuvant immunochemotherapy for esophageal cancer (with references labeled).

	Study phase	Enrolled patients	Pathological type	Clinical stage	Immune drugs	Chemotherapy drugs	Therapy cycle	Time to surgery	Primary endpoints
SIN-ICE (18)	Pilot study	23	ESCC	II–IVA	Sintilimab	Docetaxel/nab-paclitaxel Nedaplatin	3, q3w	4–6 weeks	pCR, safety
Yang et al. (19)	Pilot study	16	ESCC	II–IVA	Camrelizumab	Paclitaxel Carboplatin	2, q3w	4 weeks	pCR
Yang et al. (20)	Pilot study	23	ESCC	II–III	Camrelizumab	Nab-paclitaxel Carboplatin	2, q3w	3–6 weeks	Safety, feasibility
Shen et al. (21)	II	28	ESCC	II–IVA	Nivolumab/pembrolizumab/camrelizumab	Nab-paclitaxel Carboplatin	2, q3w	3–5 weeks	Safety, feasibility
ESONICT-1 (22)	II	30	ESCC	III–IV	Sintilimab	Nab-paclitaxel Cisplatin	2, q3w	4–6 weeks	pCR, AEs
Xing et al. (23)	II	30	ESCC	II–IVA	Toripalimab	Paclitaxel Cisplatin	2, q3w	4–6 weeks	pCR
He et al. (24)	II	20	ESCC	III–IVA	Toripalimab	Paclitaxel Carboplatin	2, q3w	4–6 weeks	Safety, feasibility, MPR
NICE (25)	II	60	ESCC	III–IVA	Camrelizumab	Nab-paclitaxel Carboplatin	2, q3w	4–6 weeks	pCR
ESONICT-2 (26)	II	20	ESCC	III–IVA	Toripalimab	Docetaxel Cisplatin	2, q3w	4–6 weeks	pCR, AEs
NIC-ESCC2019 (27)	II	56	ESCC	II–IVA	Camrelizumab	Nab-paclitaxel, cisplatin	2, q3w	6 weeks	pCR
PEN-ICE (28)	II	18	ESCC	II–IVA	Pembrolizumab	Docetaxel/nab-paclitaxel Nedaplatin	3, q3w	4–6 weeks	Safety, efficacy
TD-NICE (29)	II	45	ESCC	II–IVA	Tislelizumab	Nab-paclitaxel Carboplatin	3, q3w	4–6 weeks	MPR

ESCC, esophageal squamous cell carcinoma; pCR, pathological complete response; AEs, adverse events; MPR, major pathological response.

Based on the research methods and findings of the above clinical trials, we can obtain many associations and inspirations.

The neoadjuvant immunotherapeutic drugs for esophageal cancer are all chosen as PD-1 inhibitors, including karelizumab, pembrolizumab, nabulizumab, sindilizumab, treprostinil, and tirilizumab; neoadjuvant chemotherapeutic regimens generally choose platinum-based drugs (cisplatin, carboplatin, and nedaplatin) combined with paclitaxel analogs (paclitaxel, doxorubicin, and leucovorin paclitaxel). The neoadjuvant immune drug in this study was PD-1-inhibiting sindilizumab, which blocked the binding of PD-1 to PD-L1 and stimulated the activation of human immune cells, thus exerting cancer inhibition, and the neoadjuvant chemotherapeutic regimen was cisplatin + albumin paclitaxel, and the chemotherapy may have activated the tumor-specific T cells by facilitating the presentation of tumor antigens and destroying the immune-suppressing factors, which further enhanced the antitumor efficacy (31).

A study of lung cancer patients receiving neoadjuvant immunochemotherapy showed a higher pCR in patients receiving three to four cycles compared to those receiving one to two cycles (32). However, there are no clinical studies discussing the cycles of neoadjuvant immunochemotherapy for esophageal cancer, and most of the clinical trials have been designed with a treatment regimen of two cycles (one cycle in 21 days), and it is not yet known whether more cycles of treatment will lead to better efficacy as in lung cancer. However, the order of administration of immunotherapy and chemotherapy may affect the pCR of patients with locally advanced esophageal cancer. A phase II clinical study showed that patients with locally advanced ESCC were randomized in a 1:1 ratio to receive neoadjuvant immunochemotherapy with chemotherapy on day 1 and teraplizumab on day 3 (experimental group) or chemotherapy and teraplizumab on day 1 (control group), and the study results showed a pCR of 36% in the experimental group and 7% in the control group, suggesting that delaying teraplizumab to day 3 in neoadjuvant immunochemotherapy may result in a higher pCR rate than that of the same day (33). Thus, we can conjecture that chemotherapy followed by immunotherapy may exert better efficacy than PD-1 monotherapy. The optimal time interval between neoadjuvant immunochemotherapy and surgery is also a question worth exploring, and a retrospective study found that for patients with locally advanced ESCC treated with neoadjuvant radiochemotherapy and esophagectomy, the overall survival was similar regardless of whether the time interval between neoadjuvant radiochemotherapy and esophagectomy was long or short (33). In clinical trials, the time interval between neoadjuvant immunochemotherapy and surgery was 4–6 weeks in most cases, and the optimal time interval needs to be further explored. Referring to the research methodology of the current clinical trial, we set the patient’s neoadjuvant immunochemotherapy as two treatment cycles, receiving chemotherapy on days 1–3, sindilizumab on day 4, and chemotherapy again on day 8. Radical esophagectomy for esophageal cancer was carried out on week 6 after the completion of neoadjuvant therapy, and two cycles of adjuvant immunotherapy were initiated on week 6 after the surgery, with the same treatment regimen as before the surgery. The optimal cycle of neoadjuvant therapy use, dosing sequence, and surgical interval still need to be confirmed in large-sample randomized clinical trials.

To date, pCR and major pathological response (MPR) are the most commonly used metrics for assessing the efficacy of neoadjuvant therapy. pCR has been defined as the absence of any surviving tumor in surgically resected specimens and in all sampled lymph nodes (ypT0N0M0) (34), and MPR has been described as ≤10% of residual surviving tumor in surgically resected specimens (35). The patient in this study had negative surgical specimens and sampled lymph nodes, achieving a pCR. However, other pathological evaluation criteria can be used for esophageal cancer: tumor regression grade (TRG). The Mandard criteria, proposed in 1994 (36), were the earliest used for TRG after neoadjuvant radiochemistry for squamous esophageal cancer, and several neoadjuvant post-therapy TRG criteria, all of which provide important value for tumor prognosis (37, 38). However, there is a lack of uniform criteria for TRG grading at home and abroad, and this issue is still under intense discussion.

## Conclusion

Neoadjuvant immunochemotherapy has positive significance for the treatment of patients with locally advanced esophageal cancer, and whether neoadjuvant immunochemotherapy can replace neoadjuvant synchronous radiotherapy is a future research direction, which needs to be further verified by more reliable clinical trials.

## Data availability statement

The original contributions presented in the study are included in the article/**Supplementary material**. Further inquiries can be directed to the corresponding author.

## Ethics statement

This paper is a retrospective study, no ethical requirements are involved in the paper, all useful information about the patients has been hidden, and all information in the paper has obtained the written informed consent of the patients. The studies were conducted in accordance with the local legislation and institutional requirements. Written informed consent for participation was not required from the participants or the participants’ legal guardians/next of kin in accordance with the national legislation and institutional requirements. The information and specimens involved come from the patient’s visit, are non-invasive, and are published with the patient’s written consent. Written informed consent was obtained from the individual(s) for the publication of any potentially identifiable images or data included in this article.

## Author contributions

XL: Conceptualization, Data curation, Software, Visualization, Writing – original draft, Writing – review & editing. MW: Data curation, Investigation, Methodology, Visualization, Writing – review & editing, Writing – original draft. DM: Investigation, Software, Visualization, Writing – original draft. YT: Investigation, Software, Writing – original draft. QS: Conceptualization, Formal analysis, Methodology, Project administration, Resources, Supervision, Writing – original draft, Writing – review & editing.
