# Supplementary material for: Case report: A case study of neoadjuvant immunochemotherapy for locally advanced esophageal squamous carcinoma
Source: Front Oncol. 2024 Jul 4;14:1332314. doi: 10.3389/fonc.2024.1332314 (PMC11254663; doi:10.3389/fonc.2024.1332314)
Supplement: Supplementary file 1 [file DataSheet_1.pdf]

CARE

1 .The diagnosis or intervention of primary focus followed by the words “case report”

A case report of neoadjuvant immunochemotherapy for locally advanced esophageal squamous carcinoma.

2.2 to 5 key words that identify diagnoses or interventions in this case report, including "case report"

Esophageal cancer, neoadjuvant therapy, immunotherapy, chemotherapy, case report.

3a.Introduction: What is unique about this case and what does it add to the scientific literature?

Neoadjuvant immunotherapy combined with chemotherapy or radiotherapy is currently in the exploratory stage. The esophageal cancer patients in this study underwent neoadjuvant immunochemotherapy and achieved good therapeutic effects with no recurrence so far.

3b.Main symptoms and/or important clinical findings

Progressive dysphagia for more than 1 month

3c.The main diagnoses, therapeutic interventions, and outcomes

Thoracic segmental esophageal low-differentiated squamous carcinoma cT2N2M0 stage III, two cycles of neoadjuvant immunoche-motherapy and thoracoscopic radical esophagectomy for esophageal cancer,achieved pathological complete remission pCR.

3d.Conclusion—What is the main “take-away” lesson(s) from this case?

Neoadjuvant immunochemotherapy is of positive significance for the treatment of patients with locally advanced esophageal cancer.

4.One or two paragraphs summarizing why this case is unique (may include references)

A 73-year-old elderly female patient presented to the doctor with progressive dysphagia for more than 1 month, and was clinically diagnosed as **stage III cT2N2M0** low-differentiated squamous carcinoma of the thoracic segmental esophagus after completing relevant examinations. After two cycles of neoadjuvant therapy, the patient underwent imaging examination, and enhanced CT of the chest suggested that the extent of the lesion was significantly reduced compared with the previous one and the mediastinal lymph nodes were partially reduced, and then he underwent thoracoscopic radical esophagectomy for esophageal cancer, which reached a pathological complete remission pCR by pathological evaluation, and his postoperative stage was **ypT0N0M0 stage I**. As of today, the patient has no significant postoperative complications and remains in CR.5a.De-identified patient specific information.

5a.De-identified patient specific information

Key information about the patient was hidden in the article.

5b.Primary concerns and symptoms of the patient

Patient with esophageal cancer with multiple mediastinal lymph node metastases and difficult surgical evaluation by thoracic surgery.

5c.Medical, family, and psycho-social history including relevant genetic information

The patient was previously fit, had a history of ovarian cystectomy, denied a history of smoking and alcohol consumption, and denied a family history of genetic disorders.

5d.Relevant past interventions with outcomes.

NCCN and CSCO guidelines currently recommend neoadjuvant chemo-therapy (nCT) and neoadjuvant chemoradiotherapy (nCRT) as the standard treatment modalities for locally advanced esophageal cancer [.

therapy (nCT) and neoadjuvant chemoradiotherapy (nCRT) are currently recommended by both the NCCN and CSCO guidelines as the standard treatment modalities for locally advanced esophageal cancer [. Preoperative neoadjuvant chemotherapy has a low pCR rate of no more than 10%, and neoadjuvant chemoradiotherapy improves the pCR rate compared with neoadjuvant chemotherapy, but may increase postoperative complications such as anastomotic fistula and the 5-year recurrence rate is still as high as 40-50%.

6. Describe significant physical examination (PE) and important clinical findings

Clinical presentation: progressive dysphagia for more than 1 month, accompanied by acid reflux heartburn and chest discomfort. Examination: chest was not specific, ECOG score was 1.

7.Historical and current information from this episode of care organized as a timeline.

→2023.02.06 Diagnosisi:ESCC cT2N2M0 stage III

→2023.02.16 First cycle before surgery:cisplatin 30mg d1-3 +nab-Paclitaxel 200mg d1+sintilimab 200mg d4

→2023.03.09 Second cycle before surgery:cisplatin30mgd1-3+nab-Paclitaxel200mgd1,100mgd8+sintilimab 200mg d4

→2023.04.23 Surgery:esophagectomy

→2023.05.30 The first cycle after surgery:cisplatin 30mg d1-3 +nab-Paclitaxel 200mg d1+sintilimab 200mg d4

→2023.06.21 Second cycle after surgery:cisplatin30mgd1-3+nab-Paclitaxel200mgd1,100mgd8+sintilimab 200mg d4

8a .Diagnostic testing (such as PE, laboratory testing, imaging, surveys)

gastroscopy + pathological biopsy, chest enhanced CT, barium esophageal meal and PET-CT

8b.Diagnostic challenges (such as access to testing, financial, or cultural)

After two cycles of neoadjuvant therapy, the patient underwent imaging examination, and the enhanced CT of the chest suggested that the extent of the lesion was significantly reduced compared with that of the previous treatment for mid-esophageal cancer (2023-02-06) was significantly reduced, and the mediastinal lymph nodes were partially reduced.Partial relief (PR) was achieved as assessed according to the solid tumor efficacy evaluation criteria RECIST 1.1.

8c.Diagnosis (including other diagnoses considered)

1. Clinical diagnosis:1.thoracic segmental esophageal low-differentiated squamous carcinoma cT2N2M0 stage III 2.dysphagia.

8d.Prognosis (such as staging in oncology) where applicable

The patient's postoperative pathologic staging was ypT0N0M0 Stage I. Her prognosis was good and she is still in CR status.

9a.Types of therapeutic intervention (such as pharmacologic, surgical, preventive, self-care)

Pharmacologic: cisplatin,nab-Paclitaxel,sintilimab,surgical: thoracoscopic radical esophageal cancer surgery

9b .Administration of therapeutic intervention (such as dosage, strength, duration)

First cycle:cisplatin 30mg d1-3 +nab-Paclitaxel 200mg d1+sintilimab 200mg d4

Second cycle:cisplatin30mgd1-3+nab-Paclitaxel200mgd1,100mgd8+sintilimab 200mg d4

9c .Changes in therapeutic intervention (with rationale)

After two cycles of neoadjuvant therapy, the patient underwent imaging, with enhanced CT of the chest suggesting a significant reduction in the extent of the lesion compared with the previous one and partial reduction of the mediastinal lymph nodes, followed by thoracoscopic radical esophagectomy for esophageal cancer

10a.Clinician and patient-assessed outcomes (if available) .

Pathologic complete remission pCR was achieved after surgery and the patient had significant relief of obstructive symptoms

10b.Important follow-up diagnostic and other test results

The postoperative diagnosis was low-differentiated squamous carcinoma of the thoracic esophagus ypT0N0M0 stage I. As of today, the patient remains in CR.

10c .Intervention adherence and tolerability (How was this assessed?)

No significant side effects or postoperative complications were seen in patients.

10d .Adverse and unanticipated events

N0

11a.A scientific discussion of the strengths AND limitations associated with this case report

Neoadjuvant immunochemotherapy has positive significance for the treatment of patients with locally advanced esophageal cancer. Whether neoadjuvant immunochemotherapy can replace neoadjuvant synchronous radiotherapy is a future research direction that needs to be further verified by more reliable clinical trials.

11b.Discussion of the relevant medical literature with references.

See the paper.

11c.The scientific rationale for any conclusions (including assessment of possible causes)

See chart in text and references.

11d.The primary "take-away" lessons of this case report (without references) in a one paragraph conclusion

Neoadjuvant immunotherapy has led to an increasing diversity of neoadjuvant combination treatment modalities, among which neoadjuvant immunochemotherapy has emerged, with current clinical studies initially demonstrating its efficacy and safety.

12. The patient should share their perspective in one to two paragraphs on the treatment(s) they received

See discussion section in text.

13. Did the patient give informed consent? Please provide if requested

Yes,patient has signed informed consent for neoadjuvant therapy and consent for surgery.
